# Developing future medical educators in an Australian medical program: supervisors’ reflections on the first four years of MD Professional Project implementation

**DOI:** 10.1080/10872981.2020.1819113

**Published:** 2020-09-14

**Authors:** Michelle McLean, Carmel Tepper, Neelam Maheshwari, Victoria Brazil, Christian Moro

**Affiliations:** Faculty of Health Sciences & Medicine, Bond University, Gold Coast, Australia

**Keywords:** Medical students, undergraduate medical education, Professional Projects, scholarship, supervision, MD Program, Association for Medical Education in Europe (AMEE), Essential Skills in Medical Education (ESME)

## Abstract

**Background:**

Increasingly, professional bodies expect doctors to not only provide patient care but also educate students, trainees and patients. Few medical students, however, receive formal tuition in terms of the theory and practice of medical education. A curriculum restructure from an MBBS to a Doctor of Medicine (MD) program provided an opportunity to develop three Masters streams: Clinical research, Capstones and educational Professional Projects. This submission describes how one Australian medical school is preparing some students for their future roles as medical educators through MD Professional Projects.

**Design:**

Framed by the 12 roles of the medical ‘teacher’, most students undertaking these projects take on Resource Developer (including simulation) and Assessor roles. For those choosing resource development (excluding simulation) or assessment, the Association for Medical Education (AMEE) Student Essential Skills in Medical Education (ESME) Course is compulsory. For those choosing educational research, the ESME Course is optional.

**Outcomes:**

By December 2020, four MD cohorts will have graduated with 69 students having undertaken educational MD Professional Projects, with fifty-one completing the ESME Course. MD students have created a range of resources for the curriculum, their colleagues and the local healthcare community. In addition to the expected learning we identified additional value-added outcomes for learners (e.g. skill development), the curriculum (e.g. areas of difficulty), academic supervisors’ roles (e.g. role-modelling) and for the health care community (e.g. as expert reviewers).

**Conclusions:**

Engaging in scholarly activities such the ESME Course and developing learning resources not only provided MD students with a more in-depth theoretical knowledge in a range of clinical areas, but also developed skills that would prepare them for their future roles as medical educators. As supervisors, we identified the value these projects add to the broader health community as well as personal and professional benefits for ourselves.

## Background

Increasingly, it is being recognized that doctors have the dual role of providing care and educating students, trainees and patients [1,2]. Many professional medical bodies such as the UK’s General Medical Council [3] and the Royal College of Physicians and Surgeons of Canada [4] require doctors to ‘teach’. At various points during their medical studies and training, students learn to engage with patients in different health care contexts, ranging from large urban hospitals to primary health care and General Practice (Family Medicine) clinics in the community. Their engagement with patients is generally overseen by clinical supervisors who may be trainees (e.g. interns, residents), specialists and consultants. While many senior supervisors are academic faculty, this is not always the case such as when students or trainees choose electives abroad or select remote or rural placements. Notwithstanding, there is often a cascading model in terms of responsibility for educational supervision, i.e. interns generally show medical students the ropes, residents oversee interns while specialists and consultants are responsible for residents. As such, newly graduated interns, residents and highly experienced consultants, individually and collectively, are all responsible for creating a conducive environment in which they engage students and trainees in learning opportunities, provide feedback and are often involved in assessment.

Based on the scant literature on the inclusion of theoretical and practical pedagogical knowledge and skills in the medical curriculum, it is probable that most medical students receive little or no professional development to prepare them to become educators and supervisors in the clinical setting [5–8]. Generally, this training happens during residency [8]. Marton et al.’s [6] review of educational skills development programs for medical students identified three main approaches (i.e. peer teaching, workshops, community outreach) from five countries (UK, US, Australia, Switzerland, Germany). Those authors concluded that while there was some subjective evidence to indicate improvement in ‘teaching’ skills of medical students, objective outcomes of the initiatives were generally lacking. More recently, Yeung et al. [7] reported positive outcomes (e.g. communication skills, improved learning strategies) of a longitudinal student-as-teacher program at a Canadian medical school which involved theoretical modules, practical teaching sessions, feedback and reflective exercises.

The Association for Medical Education in Europe (AMEE) has been particularly proactive in identifying the need for medical students to graduate with the necessary pedagogical knowledge and skills [9]. Through its ASPIRE-to-Excellence Awards, AMEE encourages medical schools to involve students in the pedagogy of learning and teaching. An online Student Essential Skills in Medical Education (ESME) Course offered each January immerses students in foundational medical education principles and concepts. The 12 roles framework is used as a starting point for engaging learners in the various roles of medical educators [1] and medical students [10].

In line with the Australian trend of transforming medical programs from undergraduate degrees to graduate-entry Doctor of Medicine (MD) programs [11], in 2016, the Bond University (BU) undergraduate Level 7 MBBS degree (Australian Qualifications Framework (AQF)) [12] was reconfigured such that students would graduate with two degrees, an undergraduate Bachelor of Medical Studies (8 semesters) at the end of Year 3, followed by a Level 9 Extended (9E)Masters comprising two clinical years which includes an MD project involving ± 120 hours of work [13]. This higher educational qualification (i.e. an MD degree), requires students to develop a range of skills and values across several domains. Mainly from Year 3 onwards, students are required to collect 100 points to meet the Level 9E quality standard. Sixty (60) are collected for a range of activities such as community service, Anatomy Laboratory demonstration, near-peer clinical skills tutoring, scholarship and leadership and research modules. Forty (40) points are assigned to the MD Project (Years 4 and 5) in one of three *streams*, all of which relate to areas of future professional practice:

1. *Research* (40–50% of each cohort): Individually or in teams, students work on existing projects offered by on-site academics or by clinicians in the health care setting where students undertake clinical placements.
2. *Capstone experiences* (± 40% of the cohort): Usually in pairs, students undertake international placements in a range of communities (e.g. remote Australia, Solomon Islands, India, South Africa). Their output varies from reflective reports to clinical audits [13].
3. *Professional Projects* (15–20% of the cohort): Individually or in teams, students with an interest in medical education can develop resources that can be used in the curriculum (e.g. problem-based learning cases), by fellow medical students (e.g. formative assessment, videos) or in the health care environment (e.g. simulation cases). For some, 50% of their Professional Project involves AMEE Student ESME Course completion.

Depending on the projects offered by Professional Project supervisors, Bond University students selecting the MD Professional Project stream are generally required to produce a ‘product’ that is available for review and which can be shared and evaluated. A few others engage in medical education research. Both constitute ‘scholarship’ [14]. For the purposes of this submission, Shulman’s [15] perspective of educational scholarship is applicable: *‘For an activity to be distinguished as scholarship, it should manifest at least three key characteristics: it should be public, susceptible to critical review and evaluation, and accessible for exchange and use by other members of one’s scholarly community’.*

The educational scholarship conversation probably began with Boyer [16] writing that *‘teaching both educates and entices future scholars’*, and thus the task of ‘teachers’ with expertise is to transform and extend knowledge in ways that encourage students to be critical and creative thinkers who develop the capacity to continue learning. For Boyer, ‘teachers’ should engage in critical reflection, be adequately prepared, gather and analyse evidence of student learning, leading to the *scholarship of teaching*. Glassick and colleagues identified a series of standards and a framework by which to assess educational scholarship. These included clear goals, adequate preparation, appropriate methods, significant results, effective presentation and reflective critique [14].

This article aims to describe the development and outcomes (advertised and value-added) for the first four cohorts (three completed, one to complete in 2020) of an educationally focused Professional Project stream in a new MD Program at a small, not-for-profit Australian medical school (Bond University, Gold Coast) where the average cohort size between 2016 and 2020 has been ± 115 students.

## Outline of the projects

Early in Year 4, prospective MD supervisory teams advertise their projects, which, for Professional Projects, generally requires students to produce ‘products’ which typically need to be reviewed (e.g. by clinicians to check accuracy) and which can be evaluated by the end-user (e.g. medical students using resources or completing the student-generated formative examination). Students make an application for their stream choice via the MD e-Portfolio portal. Once assigned a project, students meet with their supervisors and develop project proposals with agreed timelines. During the next 15 months (across two academic years), they work on their projects. For Professional Projects, this often means submitting a completed draft for expert review and feedback. Once feedback has been incorporated, final versions are submitted. Any educational resources developed are made available to all medical students via an online learning platform while Multiple-Choice Questions (MCQs) created are added to the student assessment bank. Students also prepare a reflective ± 3000-word MD Project Report, providing evidence of having met the agreed outcomes. MD students also submit an abstract for a conference at which they present their work. To date, three MD cohorts have graduated, with the fourth cohort graduating in December 2020.

For the student-selected topics and the Assessment MD Professional Projects, the ESME Course is compulsory and accounts for 50% of the Project (Table 1). For other Professional Projects, the ESME Course is generally extra-curricular. Other AMEE resources underpin most projects, e.g. students undertaking the general education projects are provided with two *Medical Teacher* articles: 12 roles of the teacher [1] and the 12 + 1 roles of the student [10]. In the context of the Professional Projects, student roles are primarily those of Resource Developers (including simulation) and Assessors, with some also taking on the role of educational researchers.

**Table 1.** Educational contributions and activities of four cohorts of MD students undertaking different types of MD Professional Projects (2017–2020).

Professional Project (Author; Supervisor)	Product, process or professional development completed: Three cohorts (2017–2019)	2020 Cohort: Work in progress
General education – Students’ choice of topic (MM) 50% ESME; 50% Resource development	Eighteen (18) students developed resources that are available for students on the learning management platform or the formative assessment bank or the curriculum team (PBL cases): PBL cases: Year 1 – Melanoma; Year 2 – Inflammatory bowel disease; Hashimoto’s thyroiditis Common dermatological presentations (three resources) Investigations: Liver function, kidney function, full blood count, pulmonary function Interpreting the normal and abnormal chest X-rays (VOPPs) Abdominal imaging: Rationale, interpretation including abnormalities Case studies: Hyperthyroidism and hypothyroidism Interpreting ECGs including the underlying physiology (annotated booklet) Antibiotic selection on the wards, using case studies (VOPP) 10 x ECG MCQs *ESME Course (compulsory): 18 completed*	Six (6) students completed the 2020 ESME Course in April. Resources being developed include: Contraception, abortion + MCQs (legal aspects) Interactive Anatomy workbooks Interpreting head CTs Getting to grips with heart murmurs Neonatal screening tests Planetary health offered. A student is developing learning resources involving the 2030 Sustainable Development Goals.
Health communication 50% ESME; 50% Resource development	Two (2) 2019 students produced videos: Breaking bad news Motivational interviewing *ESME Course (compulsory): 2 completed*	No students.
Simulation-based education (VB)	Sixteen (16) students (pairs or threes) Completed a seven-week simulation rotation Wrote Bond Virtual Hospital cases, e.g. extremity trauma, miscarriage, limb ischemia, confusion and cognitive decline, perineal tear and breast lump Prepared a moulaged mannequin, e.g. lacerated radial artery, necrotising fasciitis, venous leg ulcer, skin grafts. *ESME Course (optional): 7 completed*	Two (of four) students completed the 2020 ESME Course in April.
Assessment (CT) 50% ESME; 50% Resource development	Eleven (11) students completed most of the following activities: Attended workshops to learn the principles and practices of assessment Blueprinted a Year 4 formative exam to medical rotation disciplines Generated 120 MCQs Conducted an online formative examination for Year 4 peers which was run through ExamSoft®, a cloud-based digital assessment platform. Students use personal devices (Bring Your Own Device, BYOD) to complete the unsupervised, open book but time-limited examination. Review involved providing questions, identifying correct and incorrect responses, accompanied by rationales, plus a personalised report allowing students to identify strengths and weaknesses In addition, two 2019 students conducted focus groups to garner near-peer perceptions of the value of peer-generated formative examinations. *ESME Course (compulsory): 11 completed*	Five (5) students completed the 2020 ESME Course.
Research: Pathology (NM; GW)	Four (4) students undertook the following research project comparing student perceptions of digitized *vs*. actual museum specimens: Study design for the Digital Pathology museum, plus ethics application Scholarship application (Royal College of Pathologists) Digital museum resource development for 24 organ and tissue museum specimens Generated a formative assessment to compare real museum specimens *vs*. digitized images Conducted of a pilot study, including statistical analysis	Journal article in preparation.
Research: Mixed reality (CM)	Three (3) students completed a literature review of medical databases to determine the current uses of virtual and augmented reality in Anatomy education. They also created three Anatomy learning modules that are available in the Anatomy laboratory as supplementary learning resources: Head and neck anatomy Head and neck lymphatics Breast (with surrounding tissue) lymphatics	Systematic literature review has been submitted (currently under review). 2021: Project to investigate the effectiveness of the holographic device (Microsoft HoloLens), compared with traditional lecture or tablet-based sessions.

Below is a summary of supervisors’ rationales and intended outcomes for their Professional Projects:

### Educator role: Resource developers

#### Student-selected resources: Supervisor: Michelle McLean (MM)

##### Rationale

Influenced by the need for medical students to be aware of the various roles in which they may be involved as doctors, students interested primarily in the Resource Developer role are asked to consider the following when deciding on a topic:

- An area or concept they had found difficult during their medical studies,
- A perceived content or concept ‘gap’ that became obvious once in the workplace, or
- A personally interesting aspect of clinical practice that would allow deeper engagement.

Instead of creating a resource, students can opt to complete other online health professional education courses (e.g. teaching clinical skills) in addition to the ESME Course. They can also construct MCQs for the student formative assessment bank.

#### Intended learning outcomes

- Develop a theoretical foundation and key practical skills in preparation for future ‘teaching’ responsibilities as health care professionals.
- Gain a deeper understanding in an area of medical practice.
- Apply educational design principles when developing learning resources.

#### Simulation-based education: Supervisor: Victoria Brazil (VB)

##### Rationale

Experiential learning and simulation can be effective modalities for learning in health care, but expertise is required for effective design, delivery and debriefing [17]. Projects offered are designed to provide students with foundational skills and theoretical perspectives in simulation-based education (SBE) to equip them to employ experiential learning in their future educational practice.

#### Intended learning outcomes

- Apply experiential learning theory to the practical delivery of SBE, including scenario and screen-based simulation.
- Develop cases and scenarios for the Year 3 Bond Virtual Hospital (BVH) [18].
- Select simulation modalities and appropriate technology for effective SBE delivery.
- Demonstrate skills in practical simulation delivery, i.e. moulaging, using mannequins and participating in a simulation delivery training team.
- Demonstrate (novice level) strategies for effective simulation debriefing to enhance learning through reflection.

### Educator role: Assessor

#### Assessment resources: Supervisor: Carmel Tepper (CT)

##### Rationale

Assessment is currently an inextricable part of student and professional life – graduating from medical school, in specialist training, for credentialing and for continuous professional development. There is a paradigm shift from assessment of learning, using end of instruction examinations, to assessment *for* earning using information-rich, continuous assessment that supports individual learning [19,20]. Formative assessment, designed to provide maximum feedback rather than for decisions or progression, improves learning [21,22]. It is not surprising therefore, that students constantly request practice examinations to prepare for their summative and often high stakes assessment. Students completing the assessment projects reap the advertised learning benefits of creating their own assessment questions [23,24]. They also add value to the learning of their peers and colleagues by providing feedback on examination performance and by coaching them to write MCQs.

#### Intended learning outcomes

- Apply key principles and practices associated with best practice in medical assessment to generate good quality MCQs.
- Coach peers and colleagues to write good quality MCQs.
- Work through the assessment process, i.e. create a blueprint, ‘build’ an examination, deliver the assessment online, analyze post-examination performance data and provide rich, discipline-specific feedback.

### Educator role: Research

#### Pathology resource and research: supervisors: Neelam Maheshwari (NM) and Gordon Wright (GW)

##### Rationale

Students have traditionally studied gross pathology using photographs, preserved autopsy specimens and during cadaveric dissection [25]. Much of this material is, however, not readily available as it is either housed in a secure laboratory or in a museum. Even with unrestricted access to facilities, learning might be suboptimal if specimen labelling is inadequate and if an expert is not available. Digitized pathology specimens with context (i.e. patient scenarios) would allow 24-hour ‘access’ without supervision. After developing a digital pathology museum resource with images and case studies, a team of MD students was guided through a small research project from design (prospective cross-over study) to final reporting which involved canvassing Year 2 students’ views on a digital pathology ‘museum’ *vs*. real museum specimens.

#### Intended learning outcomes

- Engage with clinically relevant pathology content by writing clinical scenarios as the context for preserved specimens.
- Undertake quantitative research, from conceptualization, through statistical analysis to reporting.
- Assess clinico-pathological correlation observational and deductive reasoning skills through developing assessment items [26].
- Develop the skills required to design web-based resources.

#### Mixed reality: Supervisor: Christian Moro (CM)

##### Rationale

Technology is advancing medical education and practice, with the potential to provide life-like representations, simulations and visualizations across a range of real-world experiences [27]. Today’s medical students will eventually practice in a technology-rich environment, which is becoming increasingly automated and three-dimensional. At the same time, hardware and software innovations are ‘disrupting’ education [28]. Technology is increasingly allowing educators to offer students supplementary resources and guided exercises to allow them to individualize their learning. This is particularly useful in undergraduate medical education, as many school-leaving students require guidance and support [29,30].

Traditionally, Anatomy is studied using two-dimensional illustrations and models. Being able to locate structures in 3D is important and virtual reality can be used to facilitate the development of this skill [19]. It makes sense therefore to engage students in virtual, augmented or mixed reality, allowing them to discover the human body beyond the two dimensions depicted in textbook illustrations and to prepare them for the technology-driven clinical workplace. The mixed reality MD projects offer students an opportunity to engage in different digital reality formats to develop learning resources for their colleagues.

#### Intended learning outcomes

- Apply educational design principles to develop learning resources.
- Be familiar with digital technologies, including the ‘backend’ of technology applications and digital resource development.

## Outcomes

Table 1 summarizes the educational activities and resources developed for four cohorts (three completed + one in progress) of MD Professional Project students (*n* = 69). Also included are ESME Course completions (*n* = 51, including the 2019–2020 cohort).

As academic supervisors reflecting on our advertised intended learning outcomes, the MD Profession Project ‘process’ over four years and the range of final ‘products’ developed, we have identified several additional outcomes that have the potential to add value not only to *students’ future professional careers*, but also the *curriculum, MD supervisors* and the broader *health professional community*.

### MD students

Besides a deeper engagement with different topics in biomedical science and medicine, e.g. Physiology, Pathology, Anatomy, Radiology, Pharmacology, those completing ESME Course or an Assessment MD Professional Project are likely to gain insight into the ‘why’ and ‘how’ of assessment by allowing them to experience assessment from the ‘other’ side. This has the potential to better prepare them for their final high-stakes examinations required to graduate as doctors. In addition, with the ESME Course being certified, it is anticipated that it will stand those who completed in good stead for future professional appointments. For example, in 2019, one of the first educational MD Professional Project graduates became an adjunct faculty member while a second from the same cohort joined as a casual clinical skills tutor and OSCE examiner.

By engaging with their various MD Professional Projects, it is hoped that students develop a range of professional skills that will assist them in their future roles as educators, evidence-based researchers and managers. Our collective reflection yielded the following:

- *Reflective practice*: Through, e.g. identifying deficiencies in knowledge, skills, concepts and producing a reflective MD report.
- *Time management*: By meeting deadlines, working to schedule, prioritizing (as their medical studies also needed to be completed).
- *Digital literacy*: By using a range of software for equipment such as X-ray scanners, Oculus rift, annotating images, developing PowerPoints or videos, statistical analysis, online assessment.
- *Critical thinking*: Through developing clinical cases and scenarios, e.g. for most of the general educational resources, SBE, pathology, assessment questions.
- *Receiving and acting on feedback*: Most ‘products’, e.g. MCQs, learning resources and MD reports are submitted as drafts to be reviewed by supervisors and clinical experts and feedback provided for improvement.
- *Academic writing skills*: By developing a proposal at the start of the Project and submitting a write-up with a literature review at the end of the Project. By also writing an abstract for the MD Conference and, for some students, through writing ethics applications and contributing to journal articles.
- *Communication skills*: Through interacting with supervisors and presenting to an audience during the MD Conference.
- *Teamwork skills*: Some projects involve pairs or small teams. Even if students worked individually on projects, they are effectively on teams with their supervisors. For the SBE projects, students were also members of multi-professional delivery teams.
- *Research skills*: The Pathology project involved collecting and analyzing data and interpreting statistics. In addition, a pair of students undertaking an Assessment Project, developed a small research project to garner students’ views on student-led formative assessment. In addition, Module 6 of the ESME Course, until recently, explored medical education scholarship.
- *Psychomotor skills*: Through moulaging mannequins and manipulating digital and virtual reality hardware.

### Curriculum

In terms of adding value to the curriculum, Professional Projects:

- *Identified important concepts or ‘gaps’ in the curriculum*: Students chose, for example, to develop resources for interpreting radiological images and laboratory tests and selecting the appropriate antibiotics, providing indirect feedback on curriculum areas requiring attention.
- *Created additional delivery modalities*: The Pathology research project involved the creation of a library of digitized pathology cases which can be accessed 24/7. The mixed reality project developed virtual reality resources that enhanced the student learning experience in Anatomy and Physiology during Years 1 and 2. In addition, these resources have been used in universities in the UK, China, the USA and New Zealand.
- *Led to a student-generated assessment question bank*: This student-run Assessment Bank is now available for all students via the Bond University learning management platform.

### Academic supervisors

As MD Professional Project supervisors, we:

- Identified the need to be role models in terms of, e.g. managing time, providing timely constructive feedback, following protocols and procedures, continuing professional development and educational scholarship.
- Developed professional relationships with students, some of whom are now professional and academic colleagues while others will become future colleagues, as medical practitioners, educators or perhaps researchers.
- Were recognized by students for our supervision. Two Professional Project supervisors (MM and VB) being awarded the inaugural MD Supervisor Award in 2018 and a Professional Project supervisor (CT) was a joint 2019 recipient.

### Health professional community

In some instances, clinical experts are required to validate the accuracy of the educational resources developed. With only a small on-site clinical faculty, adjunct faculty are identified as reviewers. Engaging off-site faculty, who are often marginalized in terms of day-to-day curriculum administration, allows them to not only to be involved in scholarly activities but also provides a window into the Bond University Medical Program.

A key element of the SBE projects is the participation in a dynamic and busy educational delivery team at Gold Coast Health and in the Bond Medical Program. This ‘legitimate peripheral participation’ is highly valued by both the MD students and the health care delivery teams, emphasizing the importance of contribution as a common key element of learning and service. With most Professional Project students developing resources that are shared with their peers and used in the wider healthcare community to potentially improve patient outcomes, this can be considered as ‘giving back’ which we hope contributes to students’ sense of community.

## Discussion

After four years of offering educationally focused MD Professional Projects, a range of learning, assessment and simulation resources have been created for use by other Bond University medical students as well as for the Gold Coast health professional community to improve patient outcomes through simulation [27,31,32]. As MD Professional Project supervisors, we identified a range of skills such as reflective practice, time management, teamwork and academic writing, that students could potentially develop in undertaking the various educational projects which would be invaluable in later professional practice. With almost half of the students completing the certified ESME Course, we anticipate that this experience and qualification will provide them with the pedagogical foundations as future medical educators.

As academic supervisors, we also identified the added value of educational Professional Projects in terms of engaging adjunct clinical colleagues from the wider local health community in the Medical Program as, for example, expert reviewers. We identified our need to be role models in terms of, for example, time management and scholarship. We also recognized that through supervision, professional relationships are developed with students, some of whom are already colleagues in clinical practice or fellow educators.

Additional areas of educational Professional Projects have been included or are currently being considered. To this end, Planetary Health was offered for the first time in 2020 and law and ethics projects are being explored.

With evidence supporting a link between effective clinical teachers and clinical competence [33], it makes sense that all medical students have an opportunity to learn about what underpins good medical education practice. We thus need to explore how best to incorporate key learning and teaching skills in the curriculum such that all medical graduates are better prepared for their future role as medical educators and perhaps educational scholars.

## Conclusions

Engaging in scholarly educational activities such as resource and assessment item development not only provides students with a deeper theoretical knowledge in different areas, but also potentially develops a range of other personal and professional skills. In addition, the ESME Course and research undertaken by some should stand them in good stead as future medical educators. Value is also added for supervisors and for the wider health care community.

## Data Availability

Datasets used within each of the studies are available by emailing the corresponding author with a reasonable request.
